# Pellino1 promoted inflammation in lung injury model of sepsis by TRAF6/ NF-κB signal pathway

**DOI:** 10.1186/s12950-021-00276-6

**Published:** 2021-02-25

**Authors:** Xiaqing Liu, Zhengfang Lin, Yufeng Xu

**Affiliations:** 1 Department of Children’s respiratory, Guangzhou Women and Children’s Medical Center, Guangzhou Medical University, Guangzhou 510120, China; 2 Departmnet of Central laboratory, Guangzhou Women and Children’s Medical Center, Guangzhou Medical University, Guangzhou 510120, China; 3 Department of Clinical biological resource bank, Guangzhou Women and Children’s Medical Center, Guangzhou Medical University, Guangzhou 510120, China

**Keywords:** Pellino1, TRAF6, NF-κB, sepsis, Lung injury

## Abstract

**Background:**

This study was designed to investigate the role of Pellino1 in lung injury model of sepsis and its anti-inflammation mechanism.

**Method:**

C57BL/6 male mice (6–7 weeks old) and Pellino1^−/−^ male mice were subjected to laparotomy followed by extracorporeal cecum mobilization and ligation. THP-1 cells were treated with 500 ng/ml of LPS for 4 h. Both mRNA and protein expression of Pellino1 was increased at time dependence in lung tissue of lung injury model of sepsis mice. Knockout of Pellino1 attenuated lung injury and inhibited inflammation of sepsis mice. While Pellino1 protein enhanced lung injury and increased inflammation of sepsis mice. Pellino1 promoted inflammation in in vitro model of lung injury by TRAF6/ NF-κB signal pathway.

**Result:**

TRAF6 inhibitor attenuated the effects of Pellino1 on inflammation and lung injury in mice of sepsis. Similarly, NF-κB inhibitor also suppressed the effects of Pellino1 on inflammation and lung injury in mice of sepsis. The activation of TRAF6 or induction of NF-κB attenuated the effects of Pellino1 on inflammation in in vitro model of sepsis. The inhibition of TRAF6 or suppression of NF-κB reduced the effects of Pellino1 on inflammation in in vitro model of sepsis.

**Conclusions:**

These results suggested that Pellino1 promoted inflammation in lung injury model of sepsis by TRAF6/ NF-κB signal pathway.

## Introduction

Acute lung injury (ALI) is a clinical syndrome of acute and diffuse inflammatory lung injury caused by various internal and external pathogenic factors, which in turn causes acute respiratory failure [1]. ALI is one of the common respiratory critical illnesses. The fatality rate of ALI is as high as 40–50 % in the intensive care unit (ICU) [2]. The etiology of ALI is complex, with unclear exact pathogenesis [3]. However, it is currently believed that the excessive activation and recruitment of inflammatory cells in the lung tissue and the uncontrolled release of inflammatory factors are the root cause of ALI, independent of its specific etiology [4]. A variety of infla mmatory cytokines are involved in the occurrence and development of ALI. Among them, IL-1β and TNF-α are the two key cytokines [4]. Because they can further trigger the secretion of other pro-inflammatory cytokines, they are also called “early responsive cytokines” [4].

TRAFs are important adaptors of TNF superfamily and Toll-like/Toll/IL-1 receptor (TIR) superfamily, which play important roles in both innate immunity and acquired immunity [5]. TRAF6 is closely associated with inflammation, bone metabolism, breast development and lymph node formation, which is also involved in the pathological mechanism of immune disorders, myeloma, acute pancreatitis, prostate cancer and etc. [6, 7]. Therefore, TRAF6 is expected to be a promising therapeutic target for relevant diseases [8].

The expression of Pellino1 is increased under stimulation of Toll-like receptor (TLR) ligands and inflammatory cytokines, thereby promoting inflammatory response by activating NF-κB [9]. Under IL-1 stimulation, IL receptor-associated kinase 1 phosphorylates Pellino1 protein and activates its E3 ligase activity, causing the expression of downstream signal transduction pathway, including NF-κB and inflammatory cytokines [10]. In addition, lipopolysaccharide can induce the binding between Pellino1 and receptor interacting protein 1 for subsequent ubiquitination through the TLR signal transduction pathway, thereby activating the IκB kinase-NF-κB signal transduction pathway and causing increased expression of proinflammatory cytokines [11]. This experiment investigated the role of Pellino1 lung injury model of sepsis and its anti-inflammation mechanism.

## Materials and Methods

### Animals model

C57BL/6 male mice (6–7 weeks old) and Pellino1^−/−^ male mice (5–6 weeks old) were housed with free access to food and water. All aspects of the animal care and experimental protocols were approved by the Guangzhou Women and Children’s Medical Center, Guangzhou Medical University Committee on Animal Care. Cecal ligation and puncture (CLP) mouse model was established for sepsis model. All mice of sepsis model were anesthetized using 50 mg/kg pentobarbital sodium (Sigma-Aldrich LLC.) and then a stump was punctured once with a 22-gauge needle to extrude a small amount of stool. The cecum was put back to the normal abdominal position and the abdomen was closed.

All mice of sham group were anesthetized using 50 mg/kg pentobarbital sodium and induced with normal saline. All Pellino1^−/−^ mice were anesthetized using 50 mg/kg pentobarbital sodium, and the subjected to induce sepsis model by CLP. All mice of sepsis model were anesthetized using 50 mg/kg pentobarbital sodium, subjected to laparotomy followed by extracorporeal cecum mobilization and ligation, and then injected with recombination Pellino1 protein (1 µg/mice).

### Histological examination

Lung tissue samples after mice sacrificed were collected and fixed with 4 % paraformaldehyde for 24 h at room temperature. Lung tissue samples fixed with paraformaldehyde were paraffin-embedded. Lung tissue samples were cut into 5 *µm* sections using a paraffin slicing machine and stained with hematoxylin and eosin. Lung tissues were observed under light microscopy (magnification, × 100; BH3-MJL; Olympus Corporation, Tokyo, Japan).

### Bioanalysis measurement

Processing, bronchoalveolar lavage (BAL), and myeloperoxidase (MPO, A044-1-1, Nanjing Jiancheng Bioengineering Research Institute) activity were performed as previously described literature [12]. After induction of sepsis, BAL fluid was cataloged and BAL optical density was measured at 540 nm using aliquots. BAL hemoglobin and platelet counts were measured in 1 mL BAL fluid as previously described literature [12]. BAL total protein concentration was determined by BCA Protein Assay. BAL immunoglobulin M (IgM) was determined according to the manufacturer’s instructions. Pulmonary microvascular permeability was measured at 620 nm and 740 nm using the Evans blue dye extravasation technique as previously described literature [12].

### Enzyme-linked immunosorbent assay (ELISA)

Proinflammatory cytokines (TNF-α, IL-6, IL-1β and IL-18 levels) in the lung tissues or conditioned media from vivo or vitro model were examined using ELISA kit (H052, H007, H002, H015, Nanjing Jiancheng Bioengineering Research Institute).

### Quantitative polymerase chain reaction (qPCR)

Total RNA was isolated from mouse using RNAiso reagent (Takara Biotechnology). Prime-ScriptTM RT detection kit (Takara Biotechnology) were performed for real-time (RT)-PCR assays using ABI Prism 7500 sequence detection system. Relative levels of the sample mRNA expression were calculated and expressed as 2-DDCt. The primers used for qRT-PCR are shown: 5′-CCATGGCAGACGATGATCCC-3′ and 5′-GTATGGAAGTGATTGTCCAT-3′.

### Western blot

Protein was harvested from lung tissue or cell samples using RIPA assay and protein concentrations were measured using BCA protein assay kit. Total proteins were separated by SDS–PAGE and transferred onto polyvinylidene difluoride (PVDF) membranes (Amersham Biosciences). Membranes were blocked with 5 % non-fat milk in TBST for 1 h at 37 °C and incubated with the primary antibodies: Pellino1 (ab199336, 1:1000, ), TRAF6 (ab33915, 1:1000), NF-κB (ab32536, 1:1000, ) and β-Actin (sc-47,778, 1:5000, Santa Cruz Biotechnology) at 4 °C over-night. Membranes were washed with TBST for 15 min followed by incubation with peroxidase-conjugated secondary antibodies (sc-2004, 1:5000, Santa Cruz Biotechnology). The membrane was developed by ECL substrate and the ChemiDoc XRS system with Image Lab software (Bio-rad).

### In vitro experiments

THP-1 cell was purchased from the Type Culture Collection of the Chinese Academy of Sciences (Shanghai, China). THP-1 cell was maintained in RPMI-1640 medium (Gibco) supplemented with 10 % fetal bovine serum (Gibco) at 37 °C and 5 % CO2. Pellino1 (5′-GGATTTATGCTGCAGGGTTTG-3′ and 5′-CGCACTATATCGAGGTTTGCTT-3′), TRAF6 (5′-GGTGGTTGAATATACTCATGA-3′ and 5′-AGCTAACCCTGAAATCGAGG-3′), NF-κB (5′-GGTTCACAGAAGACTCCAAAC-3′ and 5′-CAGCCCACTGCTATCTCTGA-3′), negative control (5′-TTCTCCGAACGTGTCACGT-3′ and 5′-TTCTCTAGAACGTGTCAT-3′), siPellino1 (5′-AUUUAUGCUGCAGGGUUUGTT-3′), siTRAF6 (5′-CCATGGCAGACGATGATCCC-3′), siNF-κB (5′-TTTGATTTTGAGAAACGGTG-3′) and negative mimics (5′-UUCUCCGAACGUGUCACGUTT-3′) were transfected into cells (1 × 106 cell/ml) using Lipofectamine 2000 (Thermo Fisher Scientific, Inc.) for 48 h. After transfection, THP-1 cell was treated with 500 ng/ml of LPS for 4 h.

### Immunofluorescent staining

After induction model, THP-1 cell was fixed with 4 % paraformaldehyde for 15 min and then incubated with using 0.25 % Triton X100 for 15 min at room temperature. THP-1 cell was incubated with Pellino1 (1:100, 31,474, Cell Signaling Technology) and TRAF6 (1:100, 8028, Cell Signaling Technology) at 4˚C overnight after blocking with 5 % BSA for 1 h. After washing with PBS for 15 min, THP-1 cell was incubated with goat anti-rabbit IgG-cFL 555 (1:100, sc-362,272, Santa Cruz Biotechnology) or anti-mouse IgG-cFL 488 antibody (1:100, sc-362,267, Santa Cruz Biotechnology) for 2 h at room temperature and stained with DAPI for 15 min and washed with PBS for 15 min. The images of THP-1 cell obtained using a Zeiss Axioplan 2 fluorescent microscope (carl Zeiss AG, Oberkochen, Germany).

### Statistical analysis

Data are presented as means ± SD. A P-value of ,0.05 was considered to be significant. Student’s t test or one-way analysis of variance (ANOVA), followed by Tukey’s post hoc test for multiple range tests were used for comparisons of data.

## Results

### The expression of Pellino1 in lung injury

**Fig. 1:**
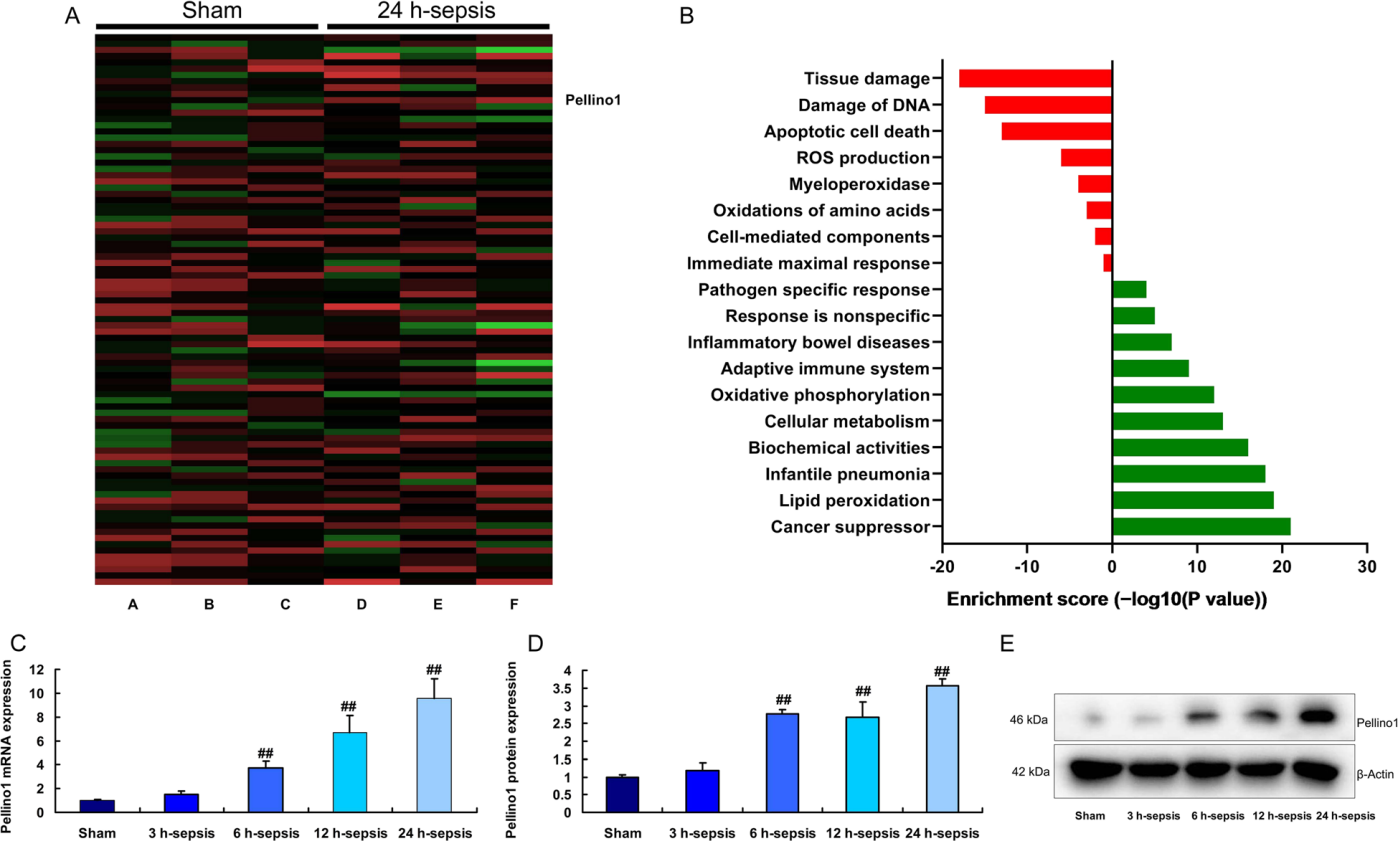
Pellino1 expression in lung injury Heat map and microarray data (**a**), Pellino1 mRNA (**b**) and protein (**c** and **d**) expressions Sham, sham control group (*n* = 6); 3 h-Sepsis, sepsis model after induction of 3 h group (*n* = 6); 6 h-Sepsis, sepsis model after induction of 6 h group (*n* = 6); 12 h-Sepsis, sepsis model after induction of 12 h group (*n* = 6); 24 h-Sepsis, sepsis model after induction of 24 h group (n = 6);. Data are presented as means ± SD using Student’s t test ##*p* < 0.01 compared with sham control group

To examine the changes of gene expression in lung tissue of lung injury model of sepsis mice, we first measured Pellino1 expression. As a result, the expression of Pellino1 was enhanced in lung tissue of lung injury (Fig. 1a and b). Both mRNA and protein expression of was increased at time dependence in lung tissue of lung injury model of sepsis mice (Fig. 1 c-e).

### Knockout of Pellino1 reduced lung injury and Pellino1 protein enhanced lung injury in sepsis mice

**Fig. 2:**
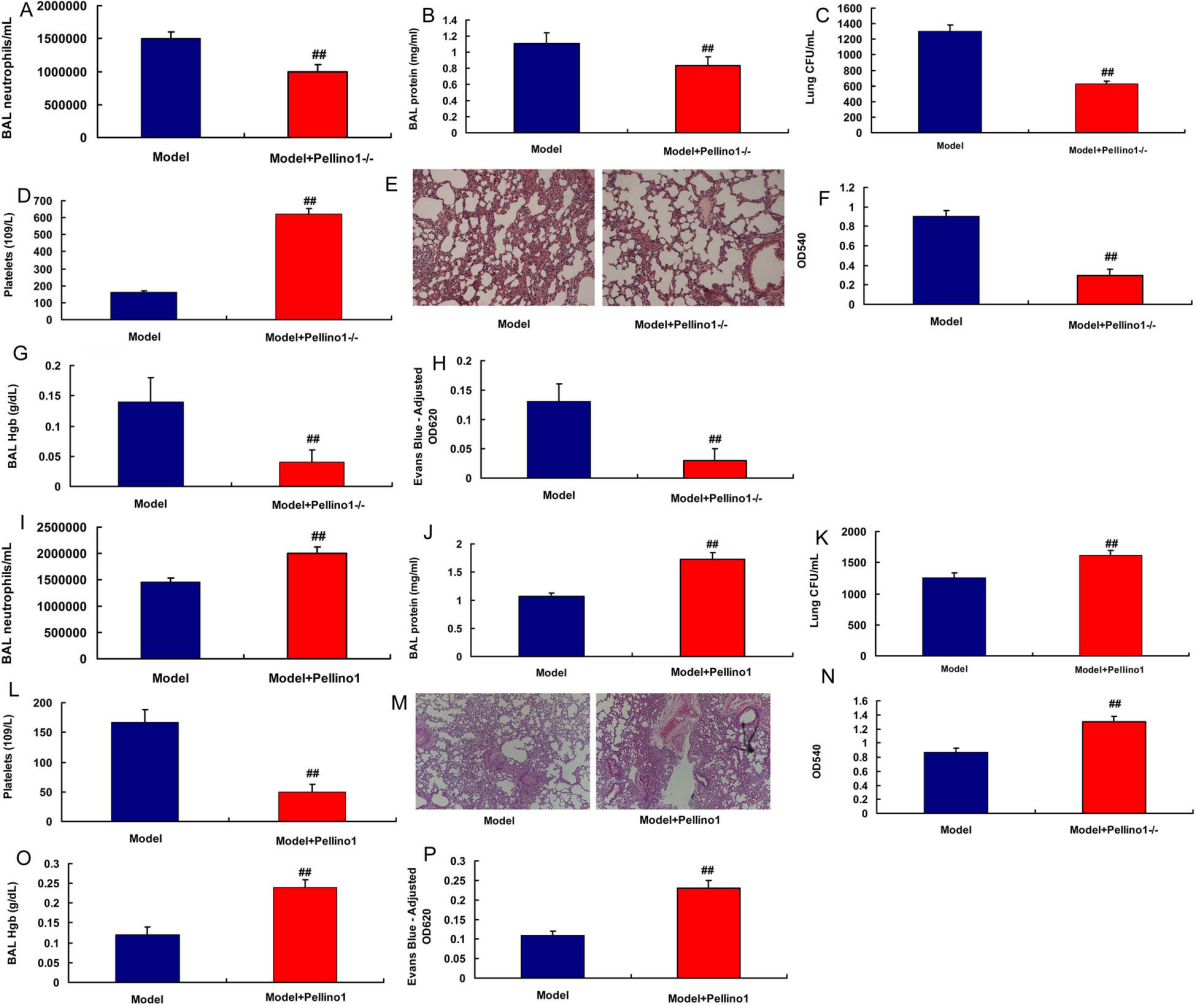
Knockout Pellino1 reduced lung injury and Pellino1 protein enhanced lung injury in sepsis mice BAL neutrophil counts/mL (**a**), BAL protein concentrations (**b**), lung bacterial CFU/mL (**c**), circulating platelet counts (**d**), lung tissue by HE assay (**e**), BAL fluid hemoglobin (**f**), BAL fluid OD540 measurements at 0 h (**g**) and Evans blue dye measurements of lung tissue homogenates at OD620 (**h**) in Pellino1^−/−^ mice;BAL neutrophil counts/mL (**i**), BAL protein concentrations (**j**), lung bacterial CFU/mL (**k**), circulating platelet counts (**l**), lung tissue by HE assay (**m**), BAL fluid hemoglobin (**n**, BAL fluid OD540 measurements at 0 h (**o**) and Evans blue dye measurements of lung tissue homogenates at OD620 (**p**) Model, sepsis model group (*n* = 6); Model + Pellino1-/-, Pellino1^−/−^ mice of sepsis model group (*n* = 6); Model + Pellino1, sepsis mice model by human recombinant Pellino1 protein group (*n* = 6). Data are presented as means ± SD using ANOVA followed by Tukey’s post hoc test ##*p* < 0.01 compared with sepsis model group

We further examined the function of Pellino1 in lung injury in sepsis mice. Consequently, we found that the inhibition of BAL neutrophil, BAL protein concentrations, lung bacterial CFU, BAL fluid hemoglobin (Hgb) concentration, lung injury, BAL fluid OD540 and lung tissue homogenates at OD620, and circulating platelet counts were increased in lung injury in Pellino1^−/−^ mice of sepsis (Fig. 2a and h).

Recombinant Pellino1 protein was used to induce lung injury in sepsis mice. As expected, recombinant Pellino1 protein increased BAL neutrophil, BAL protein concentrations, lung bacterial CFU, BAL fluid Hgb concentration, lung injury, BAL fluid OD540 and lung tissue homogenates at OD620, and inhibited circulating platelet counts in lung injury of sepsis mice (Fig. 2I and P).

### Pellino1 promoted inflammation both in vivo and vitro model of lung injury by TRAF6/ NF-κB

**Fig. 3:**
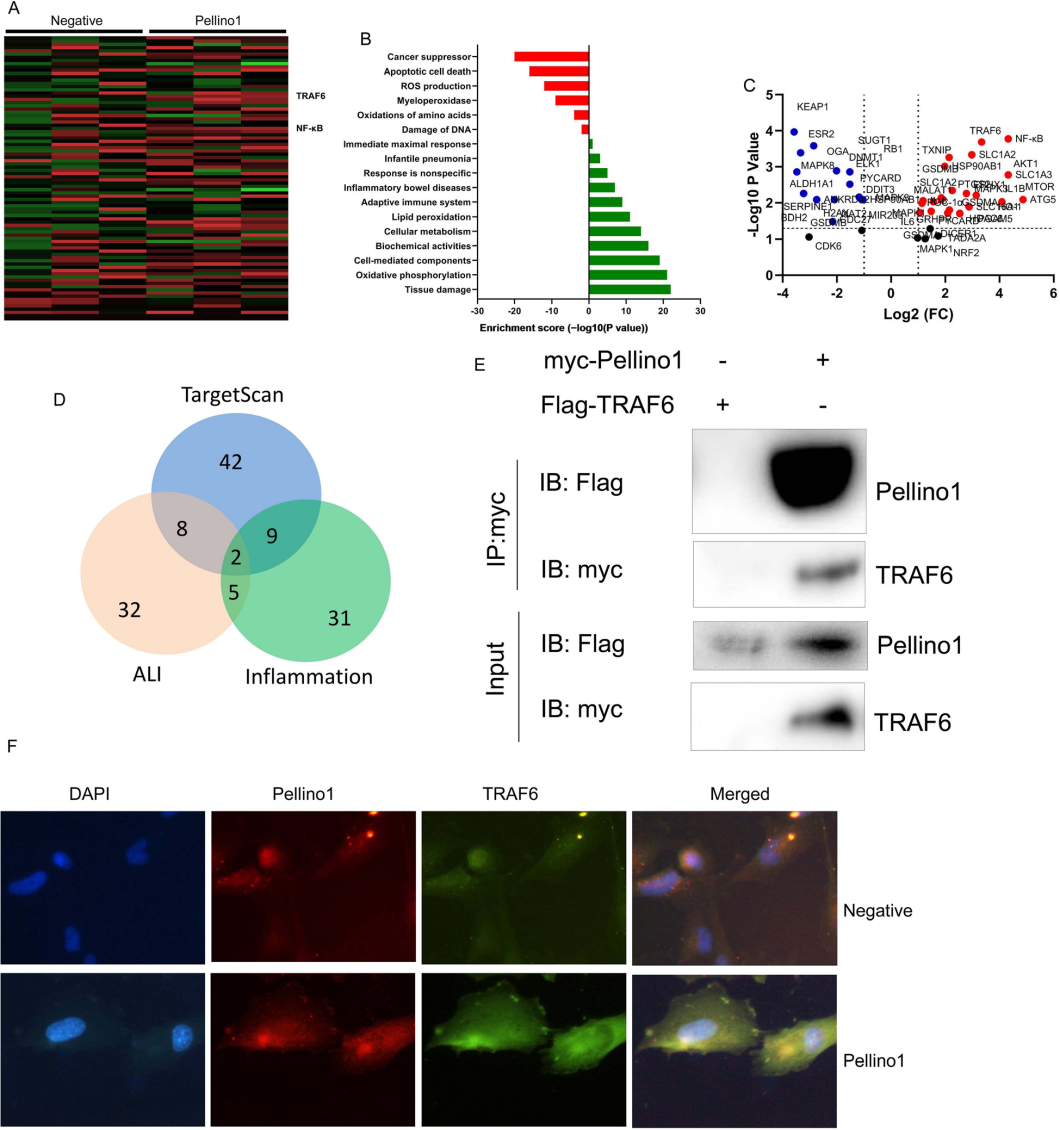
TRAF6/ NF-κB was target spot of Pellino1 in lung injury in mice of sepsis Heat map and microarray data (**a** and **b**), volcano figure (**c**), interpretation of result (**d**), Pellino1 was pulled down by TRAF6 (**e**), over-expression of Pellino1 induced TRAF6 expression (**f**) Negative, negative group (*n* = 3); Pellino1, over-expression of Pellino1 group (*n* = 3)

To define the mechanism of Pellino1 in lung injury, we analyzed the gene expression of Pellino1-regulated inflammation gene in lung injury. As a result, the expression of TRAF6/ NF-κB was up-regulated and TRAF6/ NF-κB may be a target spot of Pellino1 in vitro by over-expression of Pellino1 (Fig. 3a and d). Pellino1 was pulled down by TRAF6 in a mutual pulldown experiment using anti-Flag antibody (Fig. 3e). Over-expression of Pellino1 increased the expression of TRAF6 in vitro (Fig. 3f).

**Fig. 4:**
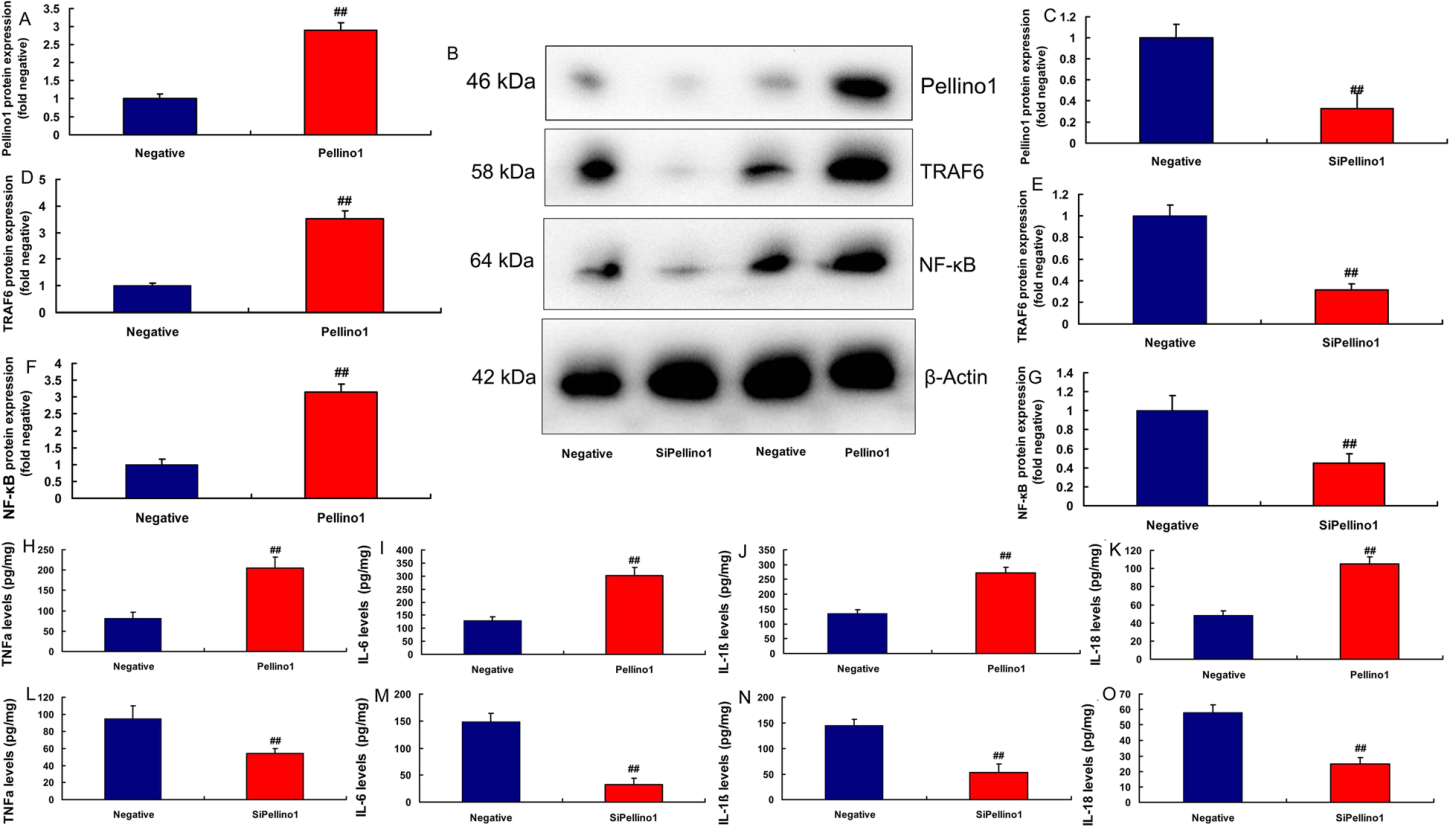
Pellino1 promoted inflammation in vitro model of lung injury by TRAF6/ NF-κB Over-expression of Pellino1 induced Pellino1/TRAF6/ NF-κB protein expressions in vitro model (**a**, **b**, **d** and **f**); down-regulation of Pellino1 induced Pellino1/TRAF6/ NF-κB protein expressions in vitro model (**b**, **c**, **e **and **g**); over-expression of Pellino1 increased TNF-α, IL-6, IL-1β and IL-18 levels in vitro model (**h**, **i**, **j** and **k**); down-regulation of Pellino1 inhibited TNF-α, IL-6, IL-1β and IL-18 levels in vitro model (**l**, **m**, **n** and **o**) Negative, negative group (*n* = 3); Pellino1, over-expression of Pellino1 group (*n* = 3); siPellino1, down-regulation of Pellino1 group (*n* = 3). Data are presented as means ± SD using Student’s t test ##*p* < 0.01 compared with negative group

Moreover, over-expression of Pellino1 induced the protein expression of Pellino1, TRAF6 and NF-κB in vitro of lung injury (Fig. 4a, b, d and f). Down-regulation of Pellino1 suppressed the protein expression of Pellino1, TRAF6 and NF-κB in vitro of lung injury (Fig. 4b c, 4e, 4g). Over-expression of Pellino1 increased the levels of TNF-α, IL-6, IL-1β and IL-18 in vitro (Fig. 4h and k). While down-regulation of Pellino1 reduced the levels of TNF-α, IL-6, IL-1β and IL-18 in vitro (Fig. 4l and o).

**Fig. 5:**
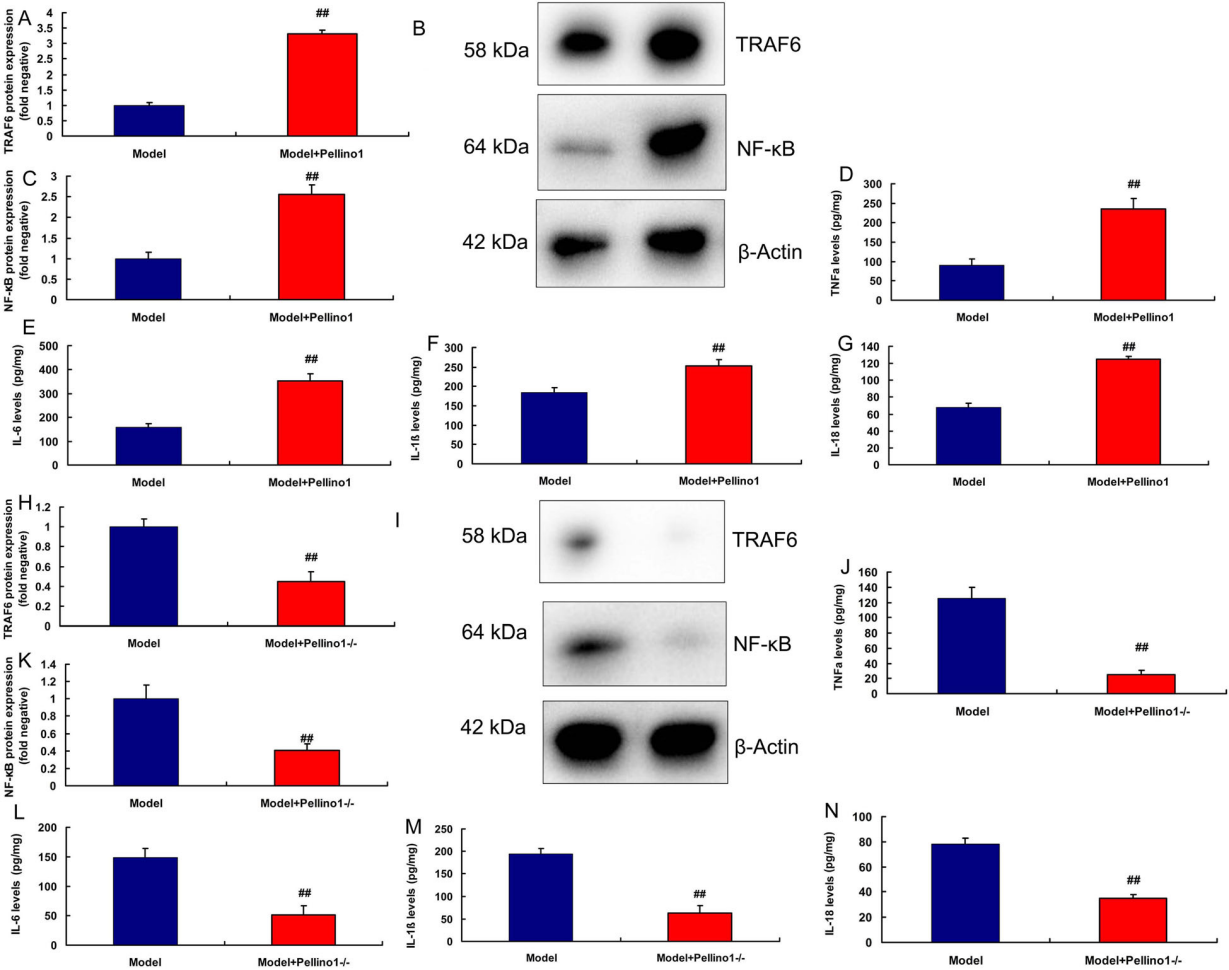
Pellino1 promoted inflammation in vivo model of lung injury by TRAF6/ NF-κB Pellino1 protein induced TRAF6 and NF-κB protein expressions and TNF-α, IL-6, IL-1β and IL-18 levels in vivo model of lung injury (**a**, **b**, **c**, **d**, **e**, **f** and **g**); TRAF6 and NF-κB protein expressions and TNF-α, IL-6, IL-1β and IL-18 levels in Pellino1-/- mice model of lung injury (**h**, **i**, **j**, **k**, **l**, **m** and **n**) Model, sepsis model group (*n* = 3); Model + Pellino1-/-, Pellino1^−/−^ mice of sepsis model group (*n* = 3); Model + Pellino1, sepsis mice model by human recombinant Pellino1 protein group (*n* = 3). Data are presented as means ± SD using Student’s t test ##*p* < 0.01 compared with sepsis model group

Next, we found that Pellino1 protein could induced the protein expression of Pellino1, TRAF6 and NF-κB and increased the levels of TNF-α, IL-6, IL-1β and IL-18 in mice of sepsis (Fig. 5a and g). Pellino1^−/−^ mice suppressed the protein expression of Pellino1, TRAF6 and NF-κB and inhibited the levels of TNF-α, IL-6, IL-1β and IL-18 in mice of sepsis (Fig. 5h n).

### The regulation of TRAF6 was involved in the effects of Pellino1 in lung injury model

**Fig. 6:**
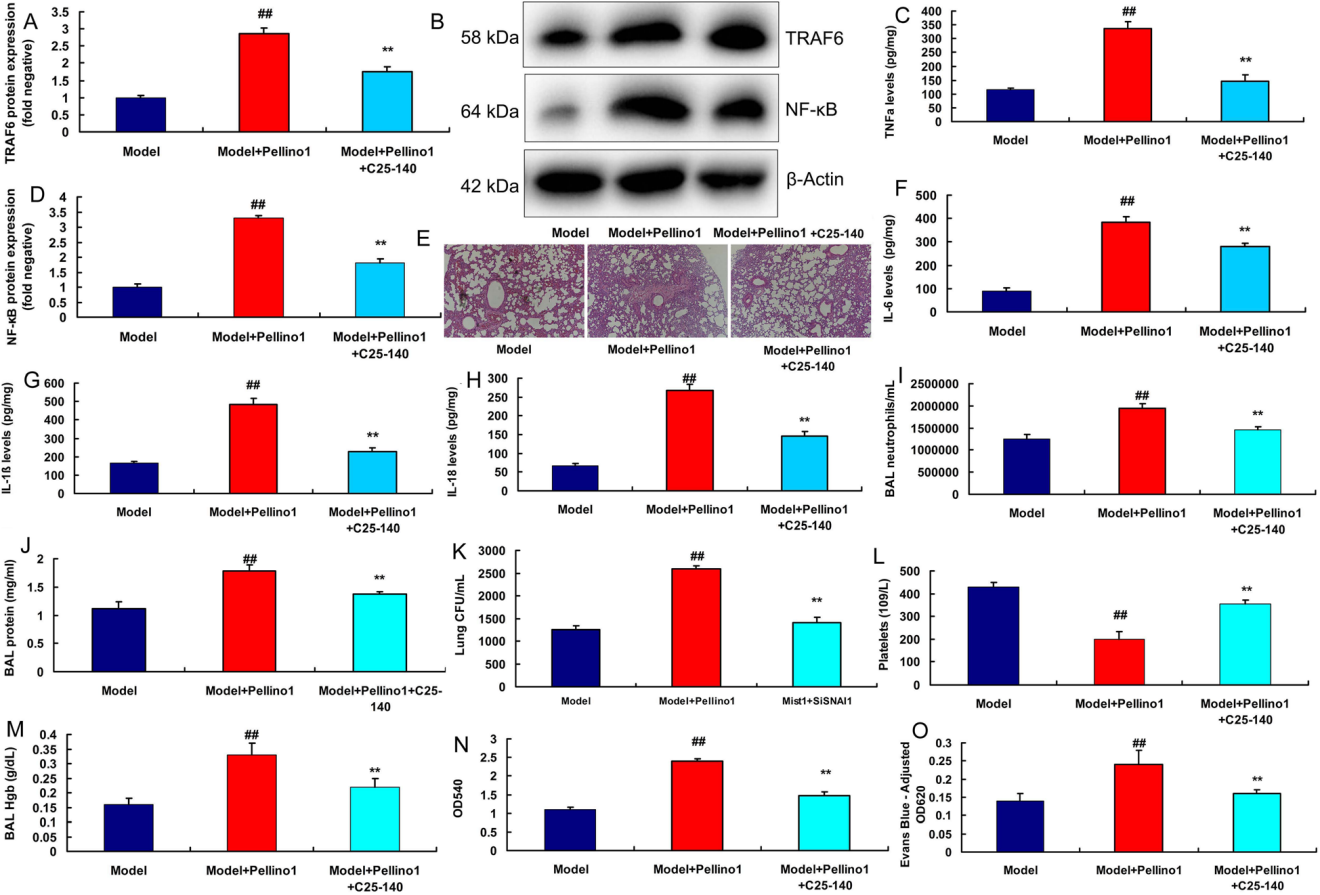
The regulation of TRAF6 participated in the effects of Pellino1 in mice model of lung injury model TRAF6 and NF-κB protein expressions (**a**, **b**, **d**), TNF-α level (**c**), lung tissue by HE assay (**e**), IL-6 levels, IL-1β and IL-18 levels (**f**, **g**, and **h**), BAL neutrophil counts/mL (**i**), BAL protein concentrations (**j**), lung bacterial CFU/mL (**k**), circulating platelet counts (**l**), BAL fluid hemoglobin (**m**), BAL fluid OD540 measurements at 0 h (**n**) and Evans blue dye measurements of lung tissue homogenates at OD620 (**o**) Model, sepsis model group (*n* = 6); Model + Pellino1, sepsis mice model by human recombinant Pellino1 protein group (*n* = 6); Model + Pellino1 + C25-140, sepsis mice model by human recombinant Pellino1 protein and TRAF6 inhibitor group (*n* = 6). Data are presented as means ± SD using ANOVA followed by Tukey’s post hoc test ##i < 0.01 compared with sepsis model group; **p < 0.01 compared with sepsis model by human recombinant Pellino1 protein group

We continued to examine the role of TRAF6 in the effects of Pellino1 in lung injury model. TRAF6 inhibitor (C25-140) could suppress the protein expression of TRAF6 and NF-κB, reduced lung injury and inhibited the levels of TNF-α, IL-6, IL-1β and IL-18 in mice treated with Pellino1 protein (Fig. 6a and h). TRAF6 inhibitor reduced BAL neutrophil, BAL protein concentrations, lung bacterial CFU, BAL fluid Hgbconcentration, lung injury, BAL fluid OD540 and lung tissue homogenates at OD620, and increased circulating platelet counts in lung injury of mice treated with Pellino1 protein (Fig. 6i and o).

**Fig. 7:**
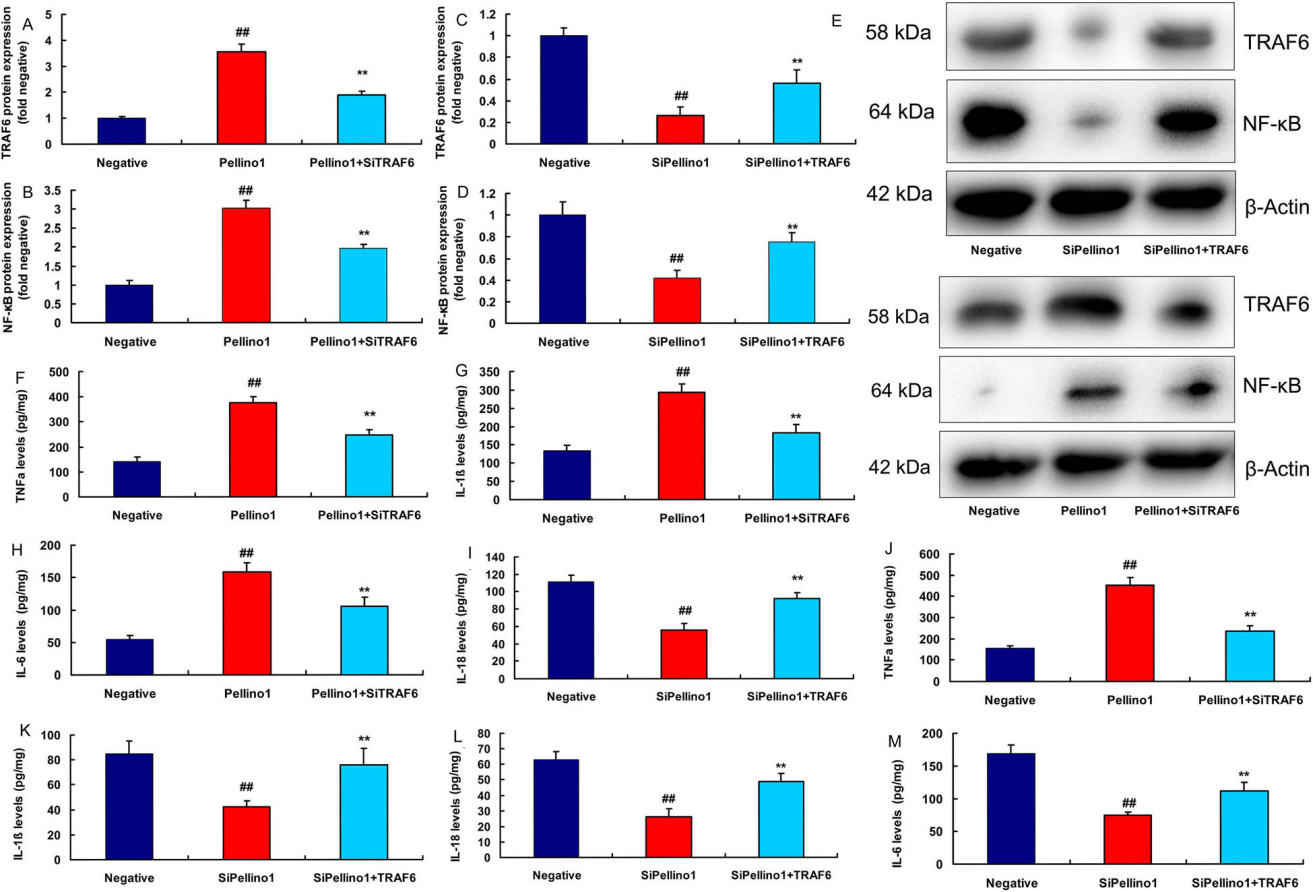
The regulation of TRAF6 participated in the effects of Pellino1 in vitro model SITRAF6 plasmid suppressed TRAF6 and NF-κB protein expressions in vitro model following over-expression of Pellino1 (**a**, **b**, **e**). TRAF6 plasmid induced TRAF6 and NF-κB protein expressions in vitro model following down-regulation of Pellino1 (**c**, **d**, **e**). SITRAF6 plasmid reduced TNF-α, IL-6, IL-1β and IL-18 levels in vitro model following over-expression of Pellino1 (**f**, **g**, **h**, **i**). TRAF6 plasmid promoted TNF-α, IL-6, IL-1β and IL-18 levels in vitro model following down-regulation of Pellino1 (**j**, **k**, **l**, **m**) Negative, negative group (*n* = 3); Pellino1, over-expression of Pellino1 group; siPellino1, down-regulation of Pellino1 group (*n* = 3); Pellino1 + siTRAF6, over-expression of Pellino1 and down-regulation of TRAF6 group (*n* = 3); siPellino1 + TRAF6, down-regulation of Pellino1 and over-expression of TRAF6 group (*n* = 3). Data are presented as means ± SD using ANOVA followed by Tukey’s post hoc test ##*p* < 0.01 compared with negative group; **p < 0.01 compared with over-expression of Pellino1 group or down-regulation of Pellino1 group

SITRAF6 plasmid suppressed the protein expression of TRAF6 and NF-κB in vitro following over-expression of Pellino1 (Fig. 7a, b and e). While TRAF6 plasmid induced the protein expression of TRAF6 and NF-κB in vitro following down-regulation of Pellino1 (Fig. 7 c-e). SITRAF6 plasmid reduced the levels of TNF-α, IL-6, IL-1β and IL-18 in vitro following over-expression of Pellino1 (Fig. 7f and i). TRAF6 plasmid promoted the levels of TNF-α, IL-6, IL-1β and IL-18 in vitro following down-regulation of Pellino1 (Fig. 7j m).

### The regulation of NF-κB was involved in the effects of Pellino1 in lung injury model

**Fig. 8:**
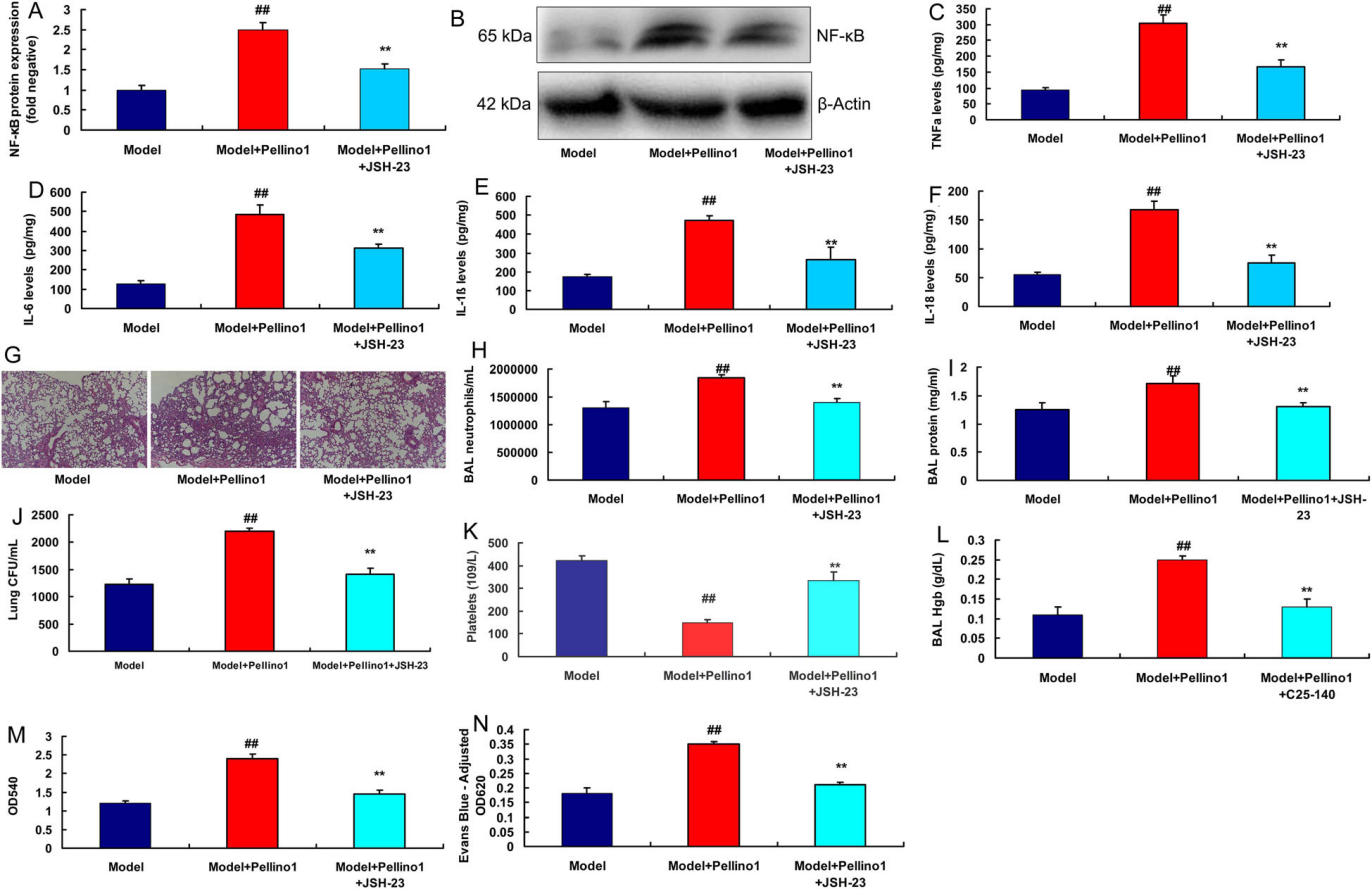
The regulation of NF-κB participated in the effects of Pellino1 in mice model of lung injury model NF-κB protein expressions (**a**, **b**), TNF-α, IL-6 levels, IL-1β and IL-18 levels (**c**, **d**, **e**, and **f**), lung tissue by HE assay (**g**), BAL neutrophil counts/mL (**h**), BAL protein concentrations (**i**), lung bacterial CFU/mL (**j**), circulating platelet counts (**k**), BAL fluid hemoglobin (**l**), BAL fluid OD540 measurements at 0 h (**m**) and Evans blue dye measurements of lung tissue homogenates at OD620 (**n**) Model, sepsis model group (*n* = 6); Model + Pellino1, sepsis mice model by human recombinant Pellino1 protein group (*n* = 6); Model + Pellino1 + JSH-23, sepsis mice model by human recombinant Pellino1 protein and NF-κB inhibitor group (*n* = 6). Data are presented as means ± SD using ANOVA followed by Tukey’s post hoc test ##*p* < 0.01 compared with sepsis model group; **p < 0.01 compared with sepsis model by human recombinant Pellino1 protein group

We further investigated the role of NF-κB n the effects of Pellino1 in lung injury model. NF-κB inhibitor (JSH-23) suppressed the protein expression of NF-κB, reduced lung injury and inhibited the levels of TNF-α, IL-6, IL-1β and IL-18 in mice treated with Pellino1 protein (Fig. 8a and f). NF-κB inhibitor decreased BAL neutrophil, BAL protein concentrations, lung bacterial CFU, BAL fluid Hgb concentration, lung injury, BAL fluid OD540 and lung tissue homogenates at OD620, and increased circulating platelet counts in lung injury of mice treated with Pellino1 protein (Fig. 8g n).

**Fig. 9:**
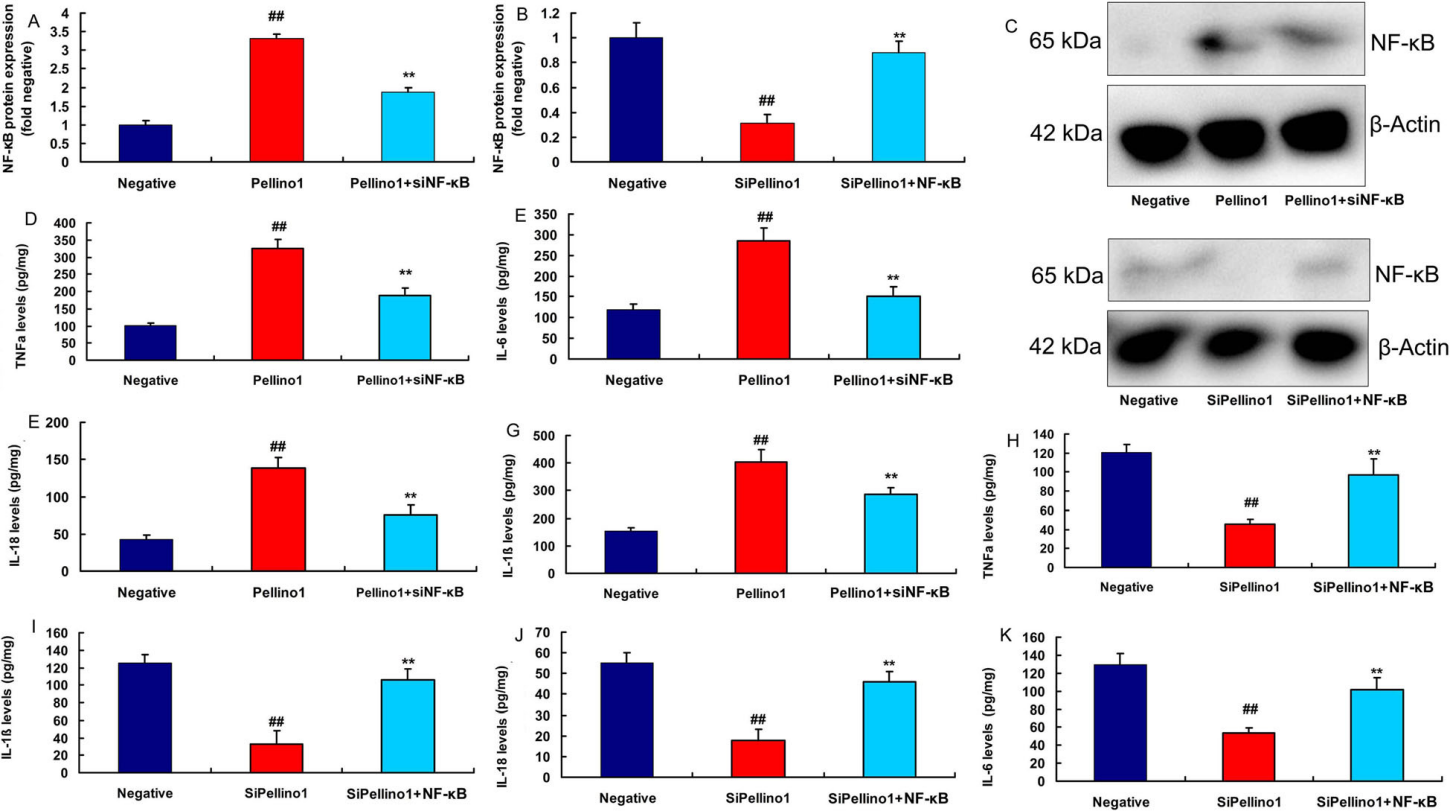
The regulation of NF-κB participated in the effects of Pellino1 in lung injury model SiNF-κB plasmid suppressed NF-κB protein expressions in vitro model following over-expression of Pellino1 (**a**, **c**). NF-κB plasmid induced TRAF6 and NF-κB protein expressions in vitro model following down-regulation of Pellino1 (**b**, **c**). SiNF-κB plasmid reduced TNF-α, IL-6, IL-1β and IL-18 levels in vitro model following over-expression of Pellino1 (**d**, **e**, **f**, **g**). NF-κB plasmid promoted TNF-α, IL-6, IL-1β and IL-18 levels in vitro model following down-regulation of Pellino1 (**h**, **i**, **j**, **k**) Negative, negative group (*n* = 3); Pellino1, over-expression of Pellino1 group; siPellino1, down-regulation of Pellino1 group (*n* = 3); Pellino1 + siNF-κB, over-expression of Pellino1 and down-regulation of NF-κB group (*n* = 3); siPellino1 + NF-κB, down-regulation of Pellino1 and over-expression of NF-κB group (*n* = 3). Data are presented as means ± SD using ANOVA followed by Tukey’s post hoc test ##*p* < 0.01 compared with negative group; **p < 0.01 compared with over-expression of Pellino1 group or down-regulation of Pellino1 group

SiNF-κB plasmid suppressed the protein expression of NF-κB in vitro following over-expression of Pellino1 (Fig. 9a c). NF-κB plasmid induced the protein expression of NF-κB in vitro following down-regulation of Pellino1 (Fig. 9b c). SITRAF6 plasmid decreased the levels of TNF-α, IL-6, IL-1β and IL-18 in vitro following over-expression of TRAF6 (Fig. 9d and g). NF-κB plasmid promoted the levels of TNF-α, IL-6, IL-1β and IL-18 in vitro following down-regulation of Pellino1 (Fig. 9h and k).

## Discussion

Due to the rapid onset and severe condition of ALI, there is still a lack of effective therapeutic methods for ALI despite accumulative therapeutic approaches for clinically critically ill patients in recent years [13]. Sepsis-related ALI is still the clinical syndrome with the highest mortality rate in ICU[14]. Therefore, we found that Pellino1 mRNA and protein expressions were increase at time dependence in lung tissue of lung injury model of sepsis mice [15, 16]. Recombination Pellino1 protein enhanced lung injury in sepsis mice by induction release of inflammation factor. Hughes et al. suggest that Pellino-1 regulated immune responses in models of inflammatory lung disease [17]. Taken together, our findings demonstrated that Pellino1 is an important regulator in lung injury of sepsis, and therefore it may serve as a novel therapeutic target for lung injury of sepsis.

TRAF6 is a member of the TNF receptor family. A large number of studies have confirmed that TRAF6 plays an important role in innate immunity and acquired immunity, cell apoptosis, stress response, and inflammation [18]. Studies have also found that the expression of TRAR6 is first decreased and then increased in lung tissues in mice with ALI [7, 19, 20]. In addition, TLR4-TRAF6 pathway is considered to be involved in inflammation-related lung injury. Moreover, TRAF6 also plays an important role in the process of ALI and inflammation [18]. We observed that Pellino1 promoted inflammation in vivo and vitro model of lung injury by TRAF6/ NF-κB signal pathway. Strickson et al. identified that Pellino1 partially restored IL-1 signaling by TRAF6 in primary macrophages [21]. Moreover, these results indicated Pellino1 regulate inflammation may participate in lung injury of sepsis.

Accumulative studies have confirmed that the activation of NF-kB plays an important role in the occurrence and development of many critical illnesses, and the activation of NF-kB is also associated with a variety of inflammatory factors [22]. As a pleiotropic regulatory factor, NF-kB is at the core of inflammation and anti-inflammation [23]. Therefore, how to selectively regulate the expression of certain target genes is particularly critical for inflammatory reaction of sepsis [24]. However, the anti-inflammatory and immune effects of NF-kB inhibitors detected so far are non-specific for inflammatory factors of sepsis [25]. Excessive inhibition of NF-kB activation would destroy the dynamic balance between pro-inflammatory and anti-inflammatory factors, which would decrease the body’s defense capabilities and further aggravate disease progression [26]. Similar to this study, we found that the activation of TRAF6 or induction of NF-κB reduced the effects of Pellino1 on inflammation in vitro model of sepsis. The inhibition of TRAF6 or suppression of NF-κB reduced the effects of Pellino1 on inflammation in vitro model of sepsis. Wang et al. suggest that Pellino1 regulates neuropathic pain through the regulation of MAPK/NF-κB signaling in the spinal cord [27]. Furthermore, we found that Pellino1 triggered TRAF6/ NF-κB signaling and enhanced inflammation in lung injury of sepsis. Collectively, these results suggest that Pellino1 may be an important regulator of inflammation in lung injury of sepsis by TRAF6/ NF-κB signaling.

## Conclusions

We demonstrated that Pellino1 mRNA and protein expressions were increase in lung tissue of lung injury of sepsis and enhanced lung injury in sepsis mice by induction TRAF6/ NF-κB signaling. Our data suggest that Pellino1 may be an ideal target for the alleviation of inflammation in lung injury of sepsis.

## Data Availability

The datasets used and/or analyzed during the current study are available from the corresponding author on reasonable request.
